# Association of biliary microflora dysbiosis with cholangiocarcinoma: a single-center study

**DOI:** 10.3389/fmicb.2025.1666272

**Published:** 2025-11-03

**Authors:** Xiangyu Wang, Xue Liu, Wang Niu, Chenyue Guan, Chunlong Liu, Jiangtao Yu, Kun Song

**Affiliations:** ^1^Department of Hepatobiliary and Pancreatic Surgery, The Affiliated Fuyang Hospital of Bengbu Medical University, Fuyang, China; ^2^Graduate School, Wannan Medical College, Wuhu, China; ^3^Department of Hepatobiliary and Pancreatic Surgery, Fuyang People’s Hospital, Fuyang, China

**Keywords:** cholangiocarcinoma, metabolomics, microbiomics, 16S rRNA, bile

## Abstract

**Background:**

Cholangiocarcinoma (CCA) is an aggressive malignancy that poses a serious threat to long-term survival. In this study, we compared the biliary microbiota and metabolomic profiles of patients with CCA and those with choledocholithiasis to identify characteristic microbial species and metabolites associated with CCA and to explore the mechanisms linking microbial dysbiosis to CCA development.

**Methods:**

A total of 25 CCA patients and 25 choledocholithiasis patients were included in the study. Bile was collected intraoperatively and analyzed using 16S rRNA sequencing and liquid chromatography-mass spectrometry (LC–MS) to investigate the correlation between specific microorganisms and metabolites by integrating microbiomics and metabolomics.

**Results:**

The abundance and diversity of microorganisms were similar between the two groups; however, their microbial compositions were significantly different. Microbial–metabolite interactions may contribute to CCA development through pathways such as inflammation, oxidative stress, and energy metabolism.

**Conclusion:**

These findings reveal a unique microbial community structure and metabolic profiles in CCA patients, providing potential microbial and metabolic markers for early CCA diagnosis. They also lay a theoretical foundation for the development of novel therapeutic strategies.

## 1 Introduction

Cholangiocarcinoma (CCA) is a highly lethal tumor of the biliary system, and its incidence and etiology vary in different regions of the world. In some areas, chronic biliary inflammation due to choledocholithiasis and primary sclerosing cholangitis (PSC) may be associated with the development of CCA. And in some endemic regions, such as Southeast Asia, infection with liver fluke is the main cause of CCA development (Sripa et al., 2018; Brindley et al., 2021). Based on its anatomical features, CCA can be classified into intrahepatic cholangiocarcinoma (iCCA), perihilar cholangiocarcinoma (pCCA), and distal cholangiocarcinoma (dCCA) (Blechacz et al., 2011). Among primary malignant liver tumors, it ranks second in incidence after hepatocellular carcinoma (HCC) (Sung et al., 2021; Valle et al., 2021). It has a poor prognosis and a low survival rate, and most patients are detected at an advanced stage The overall 5-year survival rate is only 5 to 20% (Bertuccio et al., 2019; Manzia et al., 2021). The majority of patients are diagnosed at an advanced stage and can only receive palliative care (Gutiérrez-Larrañaga et al., 2021; Kam et al., 2021).

With advances in microbial sequencing technology in recent years, researchers have detected microorganisms in the biliary system of patients with bile duct cancer, even in the absence of external intervention (Yoon et al., 2021; Moon et al., 2023). The 16S rRNA gene is a core target for microbiome studies, and its use with next-generation sequencing enables rapid, comprehensive profiling of the microecology of the human biliary tract and other body sites (Han et al., 2021). Cariati et al. (2003) using the16S rRNA gene sequencing revealed that changes in biliary microbiota increased the risk of CCA (Cariati et al., 2003; Fukuda, 2002). It has also been documented that patients with CCA showed a predominance of *Akkermansia* and *Photorhabdus* in their bile as compared to patients with pancreatic cancer (Poudel et al., 2023). Not only that, but the altered microbiota in the biliary tract can determine liver fibrosis and tumor cell proliferation through activation of the Toll-like Receptor (TLR) (Seki et al., 2007; Elvevi et al., 2023). Dysregulated gut microbes also contribute to the development of biliary systemic diseases by altering bile acid metabolism due to the presence of the enterohepatic cycle (EHC) (Dai et al., 2013; Liu et al., 2014; Shalon et al., 2023). This has raised the question of whether CCA is microbiologically related in some way.

Metabolomics technology, as an analytical tool, plays an indispensable role in elucidating disease pathogenesis and therapeutic strategies (Pang and Hu, 2023). Liquid chromatography–mass spectrometry (LC–MS), which integrates the separation capacity of liquid chromatography with the detection sensitivity of mass spectrometry, has transformed untargeted metabolomics and is now the most widely used platform in metabolomics research (Cui et al., 2018; Chen et al., 2023). Lori et al. performed LC–MS lipidomic analysis and free fatty acid quantification on human iCCA cells, demonstrating that fatty acid metabolism can alter the stemness characteristics of CCA (Lori et al., 2024). Chai et al. demonstrated that bacteria can modulate amino acid metabolic pathways in tumor patients by integrating metabolomic and transcriptomic analysis (Chai et al., 2023). Previous studies have reported that host gene products can interact with microbial metabolites, and these interactions include the regulation of host tumor immunity (Postler and Ghosh, 2017; Song et al., 2020).

Our study integrated 16S rRNA sequencing and LC–MS untargeted metabolomics to comprehensively analyze bile samples from patients with CCA and those with choledocholithiasis. This study aimed to investigate the microbiological and metabolic differences between the two diseases, identify microbial species and metabolites characteristic of CCA, and explore the mechanisms linking microbial dysbiosis to biliary tract disease. Our findings are intended to inform early diagnostic approaches for CCA and to nominate potential therapeutic targets.

## 2 Materials and methods

### 2.1 Patients enrollment and samples collection

The study adhered to the ethical guidelines of the Declaration of Helsinki and was approved by the Ethics Committee of Fuyang People’s Hospital. This study is exploratory in nature, and no formal sample size calculation was performed. The purpose of selecting the sample size was to provide preliminary data and insights for future research. We prospectively collected 25 cases of CCA (Group T) and 25 cases of choledocholithiasis (Group S) from patients who visited Fuyang People’s Hospital between August 2023 and December 2024. They were diagnosed and underwent laparoscopic surgery at Fuyang People’s Hospital. During the operation, the common bile duct was thoroughly exposed and separated, and a sterile syringe was connected to a puncture needle to collect 5 mL of bile at a certain distance from the tumor in the common bile duct, which was evenly divided into 2 portions and put into sterile bile storage tubes, and then immediately put into a cryogenic refrigerator at −80 °C to be stored for subsequent 16S rRNA sequencing and metabolomics studies, and the whole process was strictly in accordance with the principle of asepticity. Exclusion criteria were as follows: (1) incomplete basic or clinical data; (2) history of malignant tumors, chemotherapy, biliary surgery, bile duct stent placement, or gallbladder fibrosis; (3) use of antibiotics within 3 months prior to surgery.

### 2.2 DNA extraction and16S rRNA sequence project

Genomic DNA was extracted from the samples using a commercial DNA extraction kit. DNA concentration and quality were assessed using a NanoDrop 2000 spectrophotometer (Thermo Fisher Scientific, United States) and agarose gel electrophoresis. The extracted DNA was stored at −20 °C until further use. Genomic DNA served as the template for amplification with specific barcoded primers and Takara Ex Taq high-fidelity polymerase, according to the selected sequencing region. The bacterial 16S rRNA gene V3–V4 region was amplified by polymerase chain reaction (PCR) using universal primers 343F (5′-TACGGRAGGCAGCAG-3′) and 798R (5′-AGGGTATCTAATCCT-3′). PCR products were confirmed by agarose gel electrophoresis, purified with AMPure XP magnetic beads, and used as templates for a second round of PCR. The secondary PCR products were again purified with AMPure XP beads and quantified using Qubit. The concentrations were then normalized for sequencing. Sequencing was performed on the Illumina NovaSeq 6000 platform, generating 250-bp paired-end reads.

### 2.3 Metabolomics analysis of bile

A total of 50 bile samples were analyzed in this study. Samples stored at −80 °C were thawed on ice, and 100 μL aliquots were transferred into 1.5 mL EP tubes. Subsequently, 300 μL of methanol–acetonitrile (v/v = 2:1) containing an internal standard (4 μg/mL) was added, followed by vortexing for 1 min and ultrasonic extraction on ice for 10 min. The mixture was centrifuged at 12,000 rpm for 20 min at 4 °C, and 150 μL of the resulting supernatant was transferred into LC–MS vials with inserts for analysis. Metabolomic profiling was performed using a Waters ACQUITY UPLC I-Class Plus system coupled with a Thermo QE HF high-resolution tandem mass spectrometer. Quality control (QC) samples were prepared by pooling equal aliquots from all samples. QC samples were used to equilibrate the LC–MS system prior to analysis and to monitor system stability throughout the run.

### 2.4 General data and bioinformatics analysis

Clinical data, including hypertension, history of diabetes mellitus, hepatic and renal function, coagulation function, and tumor markers, were collected in as much detail as possible. Data were analyzed using SPSS Statistics version 27. For categorical variables, *p*-values were calculated using Fisher’s exact test. Continuous variables were tested for normality with the Shapiro–Wilk (S–W) test, and *p* > 0.05 was considered indicative of a normal distribution. Normally distributed data were expressed as mean ± standard deviation (SD), whereas non-normally distributed data were expressed as median and interquartile range (IQR). An independent-samples *t*-test was used to compare continuous variables between groups S and T when both followed a normal distribution. For non-normally distributed data, the Mann–Whitney U test was applied.

After the data were downloaded, the raw data sequence was first cut out of the primer sequence using Cutadapt software. Then, using DADA2 (Callahan et al., 2016), the qualified double-ended raw data from the previous step were subjected to quality control analysis, such as quality filtering, noise reduction, splicing and chimera removal according to the default parameters of QIIME2 (Bolyen et al., 2019), to obtain the representative sequences and Amplicon Sequence Variant (ASV) abundance tables.

The α-diversity analysis (Ace, Chao1, Shannon and Simpson indices) and β-diversity analysis (weighted Unifrac and Bray-Curtis distances) were performed using QIIME2 software. Differential metabolite analyses were performed using *t*-tests implemented in R software, and differential analyses of species abundance were conducted using Linear Discriminant Analysis Effect Size (LEfSe).

## 3 Results

### 3.1 Patient characteristics

A total of 50 clinical samples were included in this study, 25 cases in the group T (14 males and 11 females) and 25 cases in the group S (14 males and 11 females). NLR, IB, ALT, AST were higher in the group T than in the group S (*p* < 0.05), TB, DB, ALP, GGT, CEA, CA19-9 were significantly higher than group S, which indicated that the difference between the two groups was even more significant (*p* < 0.005), and the rest of the indices were not significantly different (Table 1).

**Table 1:** Comparison of general clinical data between two groups of patients.

Variables	S (*n* = 25)	T (*n* = 25)	*p*-value
Male (%)	14 (56)	14 (5)	1.000
Hypertension (%)	11 (44)	10 (40)	0.774
Diabetes (%)	2 (8)	1 (4)	1.000
Age (years)	71.4 ± 13.4	68.2 ± 9.9	0.349
WBC (×10^9^/L)	5.3 (4.4–6.9)	6.4 (4.5–8.2)	0.421
NEU (×10^9^/L)	3.2 (2.6–4.6)	4.1 (2.9–6.2)	0.118
LYM (×10^9^/L)	1.6 ± 0.6	1.3 ± 0.4	0.087
NLR	2.2 (1.5–3.0)	3.3 (2.5–4.6)	0.018
TB (μmol/L)	23.1 (15.9–59.6)	134 (38.7–228.4)	<0.001
DB (μmol/L)	11.5 (5.5–35.7)	109.8 (20.8–198.6)	<0.001
IBIL (μmol/L)	12.9 (8.9–19.3)	20.9 (10.4–41.1)	0.030
ALT (U/L)	56.1 (18.7–151.5)	187.7 (71.4–261.9)	0.017
AST (U/L)	35.8 (25.9–109.4)	119.2 (37.6–173.6)	0.033
ALP (U/L)	171.5 (77.4–343.6)	439.1 (225.6–642.5)	0.001
GGT (U/L)	199.3 (77.1–548.8)	517.7 (366.1–1171.5)	0.009
CEA (ng/mL)	2.7 (1.7–3.5)	4.2 (3.3–5.0)	<0.001
CA19-9 (U/mL)	12.0 (6.5–32.2)	50.3 (14.0–360.0)	0.003
BMI (kg/m^2^)	24 (21.8–25.8)	23.2 (21.9–25.2)	0.541
ALB (g/L)	37.2 ± 4.8	36.9 ± 3.2	0.797
CREA (μmol/L)	64.7 (54.6–86.8)	62.9 (53.2–72.1)	0.485
PT (S)	11.9 ± 1.2	11.7 ± 1.3	0.446
APTT (S)	27.8 (26.0–29.1)	26.8 (24.5–30.4)	0.741

NEU, neutrophil count; LYM, lymphocyte count; NLR, NEU/LYM; TB, total bilirubin; DB, direct bilirubin; IBIL, indirect bilirubin; ALT, alanine aminotransferase; AST, aspartate aminotransferase; ALP, alkaline phosphatase; CEA, carcinoembryonic antigen; CA19-9, carbohydrate antigen 19-9; BMI, Body mass index; ALB, albumin; CREA, creatinine; PT, prothrombin time; APTT, activated partial thromboplastin time.

### 3.2 Microbial diversity analysis of two groups of samples

**Figure 1:**
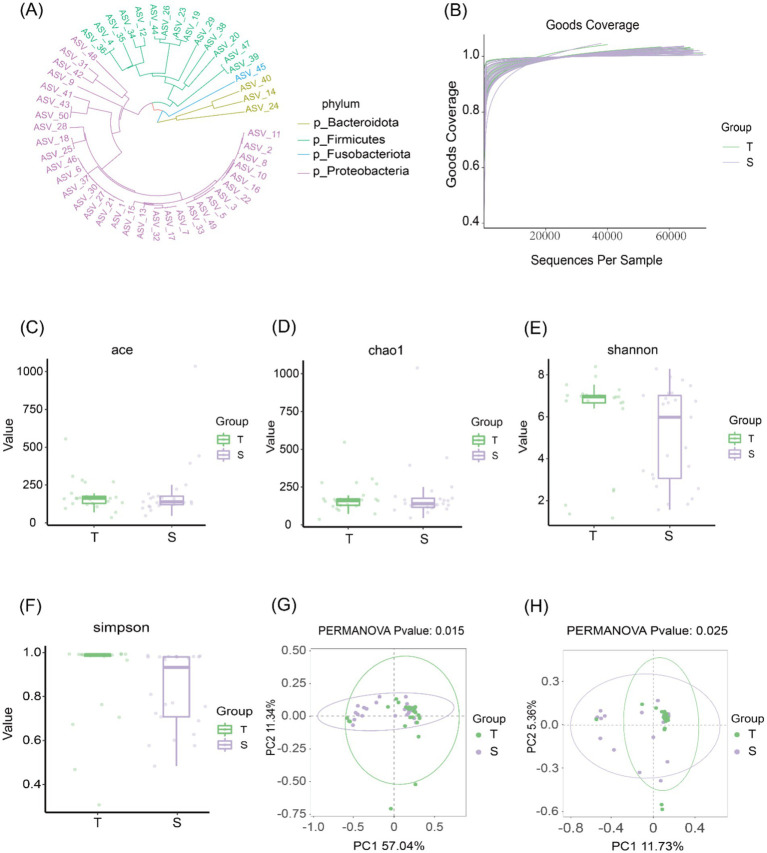
Biliary microbiological characteristics of group T and group S. **(A)** Phylogenetic tree of the top 50 ASVs at the phylum level. **(B)** Species accumulation curves showing that the curves plateau as sequencing depth increases, indicating that most microbial diversity in the samples was captured. **(C–F)** *α*-diversity analyses: Chao1 and ACE indices were used to assess community richness, while Shannon and Simpson indices were used to assess community diversity. **(G,H)** β-diversity analyses based on weighted UniFrac (*p* = 0.015) and Bray–Curtis (*p* = 0.025) distances. Each point represents one sample; points of the same color belong to the same group. Closer clustering of samples within the same group and greater separation between groups indicate distinct community structures.

A total of 3,887 differential ASVs were identified. The abundance of each ASV was calculated, and the 50 most abundant ASVs were used to construct a phylogenetic tree (Figure 1A). The four most dominant taxa were *Bacteroidetes*, *Firmicutes*, *Clostridium*, and *Proteobacteria*. The goods_coverage rarefaction curve approached a plateau with increasing sequencing depth, indicating that the sequencing coverage was sufficient to capture most of the microbial diversity in the samples (Figure 1B).

Community richness was evaluated using the Chao1 and ACE indices, and community diversity was assessed using the Shannon and Simpson indices. No statistically significant differences were observed between the two groups in Chao1 or ACE indices (*p* > 0.05), nor in Shannon or Simpson indices (*p* > 0.05), indicating comparable microbial richness and diversity between groups (Figures 1C–F). β-diversity was further assessed using weighted UniFrac and Bray–Curtis distances. Principal coordinate analysis (PCoA) revealed significant differences in β-diversity between the two groups (Figures 1G,H), suggesting distinct microbial community structures in choledocholithiasis and CCA.

### 3.3 Community structure analysis of two sample groups

**Figure 2:**
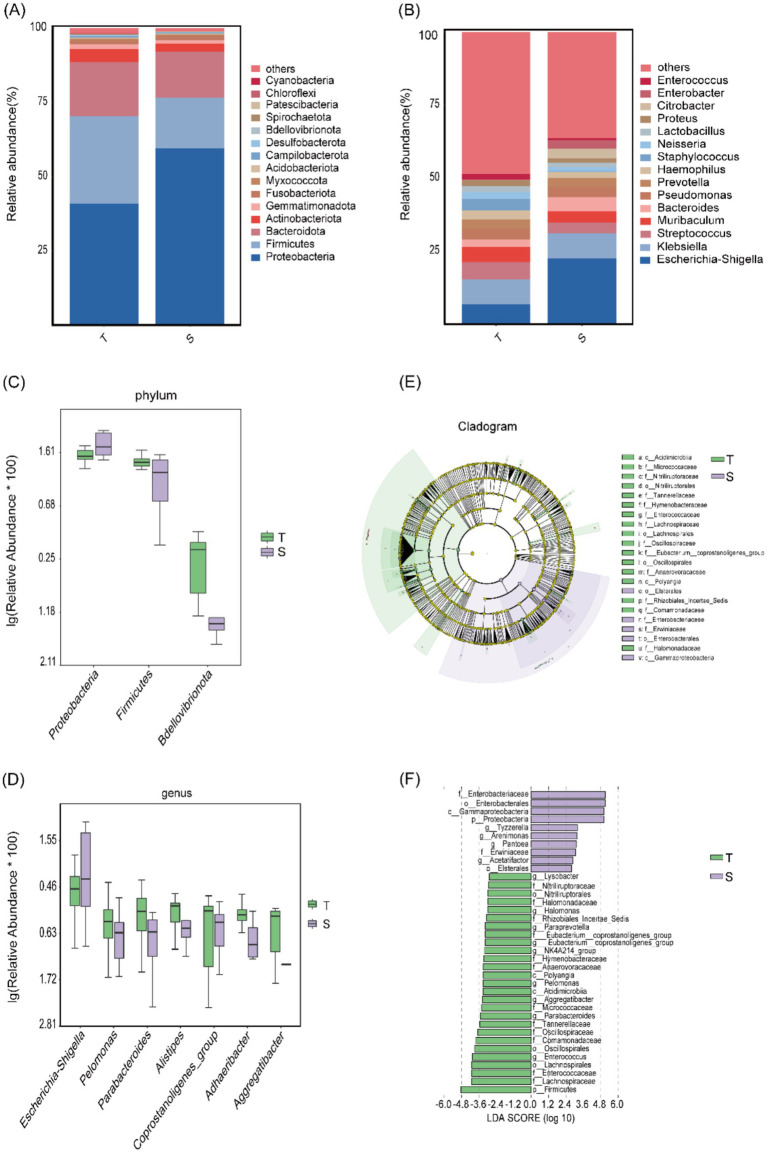
Microbial community structure analysis of group T and group S. (**A**,**B**) Microbial community composition at the phylum and genus levels. (**C,D**) Taxa showing the most significant differences at the phylum and genus levels. (**E,F**) LEfSe analysis: **(E)** Cladogram showing differentially abundant taxa; light green represents group T, light purple represents group S, and yellow nodes indicate taxa without significant differences. **(F)** LDA score plot showing taxa with relatively higher abundance in each group; light green indicates group T and light purple indicates group S.

The microbial community structure was further characterized. At the phylum level, 28 phyla were identified. Analysis of the 15 most abundant phyla showed that in group T, the dominant phylum was *Proteobacteria* (41.0%), followed by *Firmicutes* (29.4%) and *Bacteroidetes* (18.2%). Group S displayed a similar composition, with *Proteobacteria* accounting for 59.6%, followed by *Firmicutes* (17.2%) and *Bacteroidetes* (15.4%) (Figure 2A). At the genus level, 500 genera were identified. Among the 15 most abundant genera, the predominant taxa in group T were *Klebsiella* (8.6%), *Escherichia–Shigella* (6.9%), and *Streptococcus* (5.9%), whereas in group S, the most common genera were *Escherichia–Shigella* (22.7%), *Klebsiella* (8.5%), and *Bacteroidetes* (4.8%) (Figure 2B).

Using *t*-tests, we found significant differences in phylum-level abundances between groups T and S for *Firmicutes* (*p* = 0.011), *Proteobacteria* (*p* = 0.013), and *Bdellovibrionota* (*p* = 0.021). *Proteobacteria* was enriched in group S, whereas *Firmicutes* and *Bdellovibrionota* were enriched in group T (Figure 2C). At the genus level, significant differences were observed for *Parabacteroides* (*p* = 0.011), *Adhaeribacter* (*p* = 0.014), *Pelomonas* (*p* = 0.034), *coprostanoligenes_group* (*p* = 0.0383), *Escherichia–Shigella* (*p* = 0.0384), *Aggregatibacter* (*p* = 0.046), and *Alistipes* (*p* = 0.049). All genera except *Escherichia–Shigella* were enriched in group T, whereas *Escherichia–Shigella* was enriched in group S (Figure 2D).

Subsequently, LEfSe analysis (*p* < 0.05) identified 37 differentially abundant taxa across the phylum, class, order, family, and genus levels. Ten taxa were enriched in group S, including *Proteobacteria*, *Gammaproteobacteria*, *Enterobacterales*, *Erwiniaceae*, and *Tyzzerella*, whereas 27 taxa were enriched in group T, including *Firmicutes*, *Polyangia*, *Lachnospirales*, *Nitriliruptoraceae*, and *Paraprevotella* (Figures 2E,F).

### 3.4 Prediction of microbial metabolic function and metabolomics analysis

**Figure 3:**
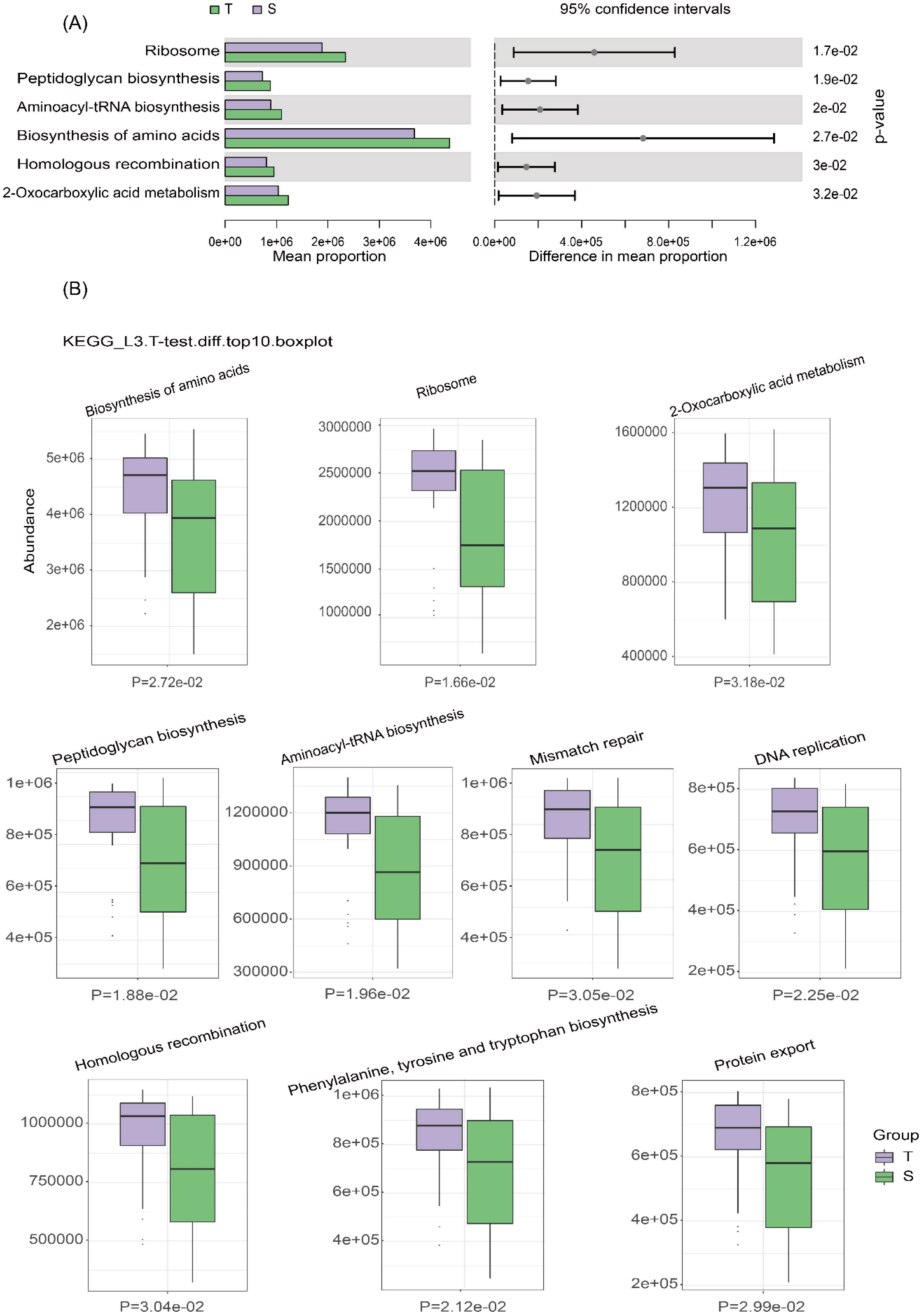
Prediction of microbial metabolic function. **(A)** Bar plot of KEGG pathway differential abundance, with bars representing the average abundance of pathways in each group. **(B)** Box plot of KEGG differential abundance results, with light purple representing group T and light green representing group S.

16S rRNA-based functional prediction was performed using the Kyoto Encyclopedia of Genes and Genomes (KEGG) database and PICRUSt2 software. Differential analysis of KEGG pathways at level 3 was conducted using a *t*-test (Figure 3A), identifying 408 metabolites, with all of the top 10 differential metabolites enriched in group T (Figure 3B). A heatmap illustrates the clustering of the top 15 differential metabolites across all samples (Supplementary Figure 1). Similarly, 16S rRNA-based COG functional prediction showed that all of the top 10 differential metabolites were enriched in group T (Supplementary Figures 2A,B).

**Figure 4:**
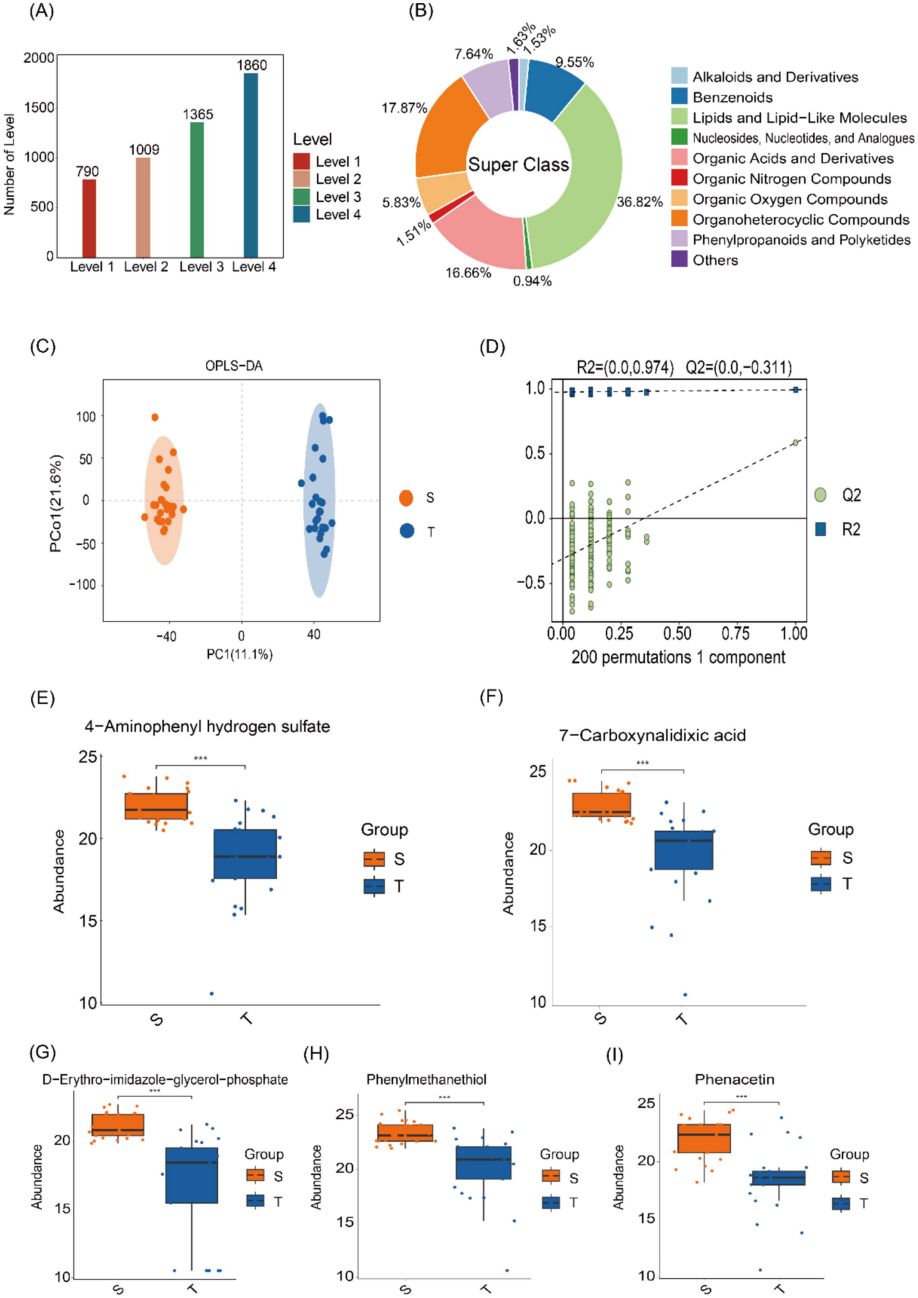
Microbial metabolomics analysis. **(A)** Distribution of metabolites across different levels. **(B)** Classification of HMDB compounds, revealing the predominance of lipids and lipoid molecules at the superclass level. **(C)** OPLS-DA score plots showing distinct metabolite profiles between the two groups. **(D)** Model validation using a permutation test, with the horizontal axis representing permutation retention and the vertical axis showing *R*^2^ and *Q*^2^ values. False lines indicate the regression of *R*^2^ and *Q*^2^. **(E–I)** Box plot displaying the distribution of differential metabolites, with significantly higher levels in group S compared to group T.

Differential metabolite studies were performed using LC–MS untargeted metabolomics, and a total of 5,024 metabolites were detected at four levels (Figure 4A). Subsequently, species classification statistics were performed at the superclass level, lipid and lipoid molecular components were found to be the most prominent, accounting for 36.82% of the total (Figure 4B). Orthogonal Partial Least Squares Discriminant Analysis (OPLS-DA) was next used to distinguish the overall differences in metabolic profiles between groups to identify the different metabolites, and the OPLS-DA scores were plotted according to the Variable Importance in Projection (VIP) values of each metabolite (Figure 4C). To assess potential model overfitting, we performed 7-fold cross-validation and 200 response permutation tests (RPTs). As shown in the figure, the model demonstrated good discrimination between the two groups (*Q*^2^ = 0.583, R^2^Y = 0.992) (Figure 4D).

Next, KEGG enrichment analysis was visualized using bubble plots, highlighting the 20 pathways with the smallest *p*-values (Supplementary Figure 3). These included adrenergic signaling in cardiomyocytes, sulfur relay system, pantothenate and CoA biosynthesis, glutathione metabolism, and histidine metabolism. Box plots display the top 50 differentially abundant metabolites ranked by *p*-value, including 4-aminophenyl hydrogen sulfate, D-erythro-imidazole-glycerol phosphate, 7-carboxynalidixic acid, phenacetin, and phenyl-methanethiol were enriched in group S (Figures 4E–I).

### 3.5 Combined analysis of microbiology and metabolomics

**Figure 5:**
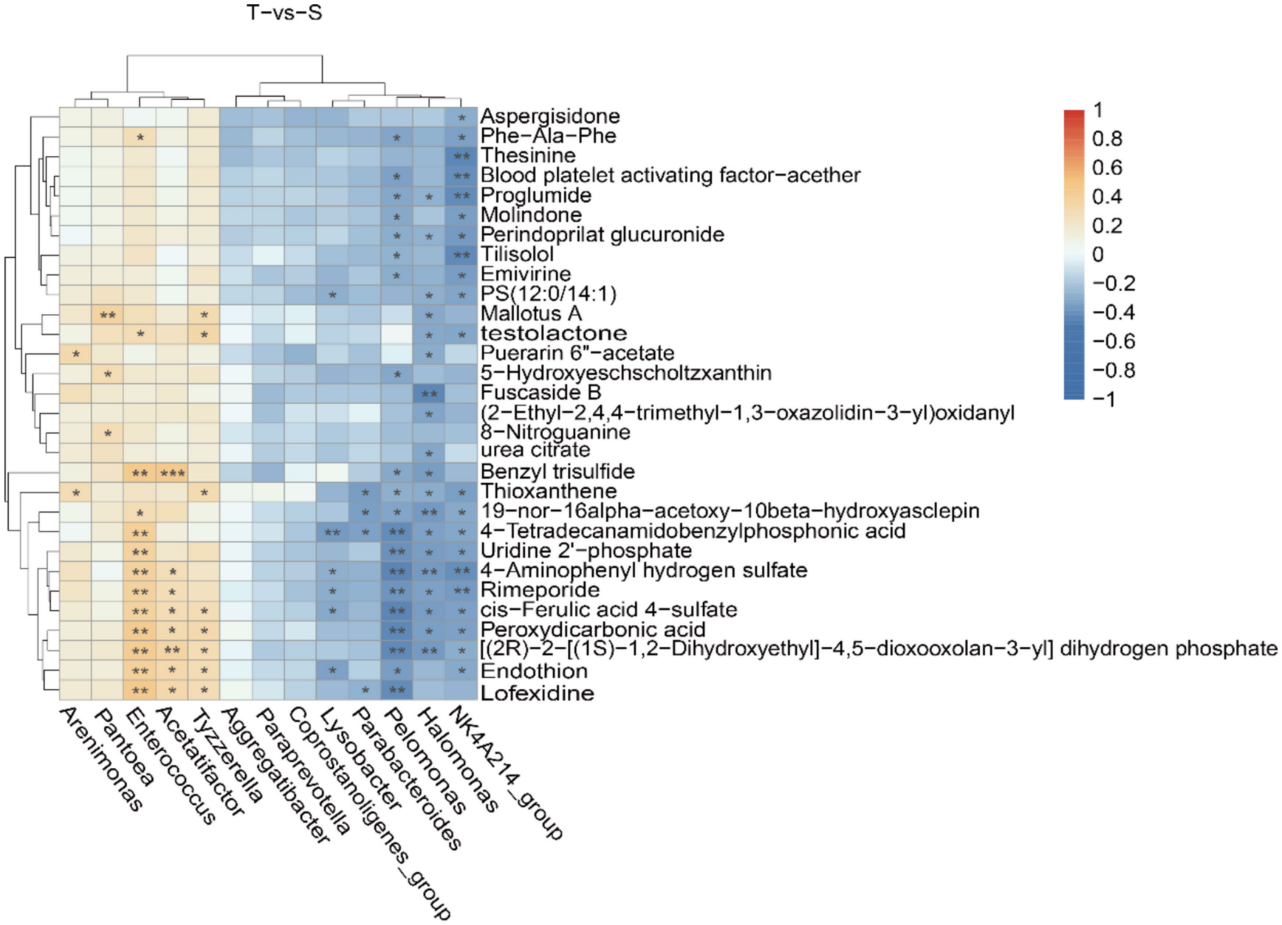
Combined microbiological and metabolomic analyzes. Correlation heatmap of the top differentially abundant microbiota and metabolites. Positive correlations are indicated in red, while negative correlations are shown in blue. *, **, and ***indicate *p* < 0.05, *p* < 0.01, and *p* < 0.001, respectively.

Based on 16S rRNA sequencing and LC–MS results, an integrated analysis was performed to investigate the relationship between microbial composition and metabolite profiles. The top 30 significantly altered microbiota and metabolites were selected, and their correlations were assessed using Spearman’s correlation coefficient (Figure 5). In group T, microorganisms such as *Pelomonas*, *Halomonas*, and *NK4A214_group* showed significant correlations with metabolites including lofexidine, peroxydicarbonic acid, and rimeporide. In group S, *Acetatifactor* was significantly correlated with benzyl trisulfide. In addition, a scatter plot demonstrated a strong positive correlation between chenodeoxycholic acid and *Enterococcus* (*R* = 0.727, *p* < 0.001) (Figure 4).

## 4 Discussion

With the advent of 16S rRNA gene sequencing, interest in the biliary microbiota in CCA has surged, challenging the long-held view that the healthy biliary tract is sterile. Although the specific pathogenetic mechanisms remain incompletely defined, accumulating evidence indicates that microorganisms play a pivotal role in CCA development.

Previous studies have investigated the role of *Helicobacter pylori* in CCA development and found that it may promote CCA by inducing inflammation and proliferation of biliary epithelial cells. Notably, bile from patients with CCA contained higher levels of DNA from *Helicobacter pylori* strains that are cagA-positive (CagA+) than bile from patients with choledocholithiasis (Boonyanugomol et al., 2012).

The concept of the “gut-liver axis” has received much attention due to its specific anatomical location (Pabst et al., 2023). There is a bidirectional liver–gut axis: metabolites and cytokines produced by the intestinal microbiota reach the liver via the portal vein, while bile acids and liver-derived metabolites, in turn, shape the intestinal microbial ecosystem (Lederer et al., 2025). In a healthy intestine, an intact intestinal barrier maintains microbial homeostasis and prevents uncontrolled absorption of luminal molecules. However, various pathological factors can disrupt the intestinal barrier, permitting bacterial translocation and contributing to the development of intestinal and hepatic disorders. Turner et al. have reported that disruption of the intestinal barrier is associated with the pathogenesis of inflammatory bowel disease (IBD) (Turner, 2009). In the same vein, there have been pointed out the role of intestinal dysbiosis in non-alcoholic fatty liver disease (Albhaisi et al., 2020). Experimental studies in colitis mice have shown that impaired intestinal barrier function leads to enrichment of gut-derived bacteria and lipopolysaccharides. Triggers Toll-like receptor (TLR) signaling in hepatocytes with induction of CXCL1 and accumulation of CXCR2^+^ polymorphonuclear myeloid-derived suppressor cells (PMN-MDSCs), and thereby promotes the development of CCA (Zhang et al., 2021). Dapito et al. previously demonstrated, in animal experiments, that gut microbiota contribute to TLR4-dependent promotion of (HCC) (Dapito et al., 2012).

16S rRNA gene sequencing has been widely used in microbiome research, and this study focused on the microbial composition and metabolism of CCA and choledocholithiasis. Liver function tests and tumor markers were significantly elevated in group T, indicating more severe hepatic injury. This likely reflects impaired bile excretion secondary to chronic biliary obstruction caused by the tumor. Comparison between the two groups revealed no significant differences in *α*-diversity, which reflects microbial richness and diversity, whereas β-diversity, which reflects microbial community composition and structure, differed significantly. Consistent with previous studies, Dai et al. investigated the microbial community structure in bile from patients with giant common bile duct stones and those with normal-sized stones. They reported comparable microbial richness between the two groups but significant differences in community composition and distribution (Dai et al., 2023). And similar results were found in the other two cohorts (Xiao et al., 2024; Park et al., 2025). At the phylum level, both groups were dominated by *Proteobacteria*, *Firmicutes*, and *Bacteroidetes*. *Proteobacteria* was the most abundant phylum in both groups, consistent with the findings of Chen et al. (2019). In addition, a previous study reported elevated levels of *Proteobacteria* in the bile of patients with choledocholithiasis and hypothesized that *Proteobacteria* may contribute to stone formation by modulating bile acid metabolism (Kakiyama et al., 2013). At the genus level, *Klebsiella* was most abundant in the group T, whereas *Escherichia–Shigella* predominated in the group S. *Klebsiella* has previously been detected in a variety of tumors, including pancreatic cancer (Matsukawa et al., 2021) and esophageal cancer (Shen et al., 2021).

In another cohort study, Cong et al. reported that enrichment of *Klebsiella* in patients with postoperative colorectal cancer was strongly associated with infectious diseases, such as *Staphylococcus aureus* infection, and was also linked to lymphatic invasion (Cong et al., 2018). Saab’s team analyzed the bile microbiota profiles of patients with extrahepatic cholangiocarcinoma (eCCA) and found that five microorganisms, including *Klebsiella* were the most predominant flora (Saab et al., 2021). However, the underlying mechanisms linking *Klebsiella* to CCA remain unclear. Our study found that *Klebsiella* was enriched in patients with CCA, and we hypothesize that it may play an important role in CCA development, which warrants further investigation. However, a subsequent study by Saab et al. (2021) reported no significant difference in *Proteobacteria* between CCA and controls, and a lower abundance of *Firmicutes* in CCA versus controls—findings that contrast with our results. In our cohort, *Proteobacteria* predominated in the group S, whereas *Firmicutes* and *Bdellovibrionota* were enriched in the group T. Further studies have shown that *Firmicutes* include several genera, such as *Trichospiridae*, *Clostridia*, and *Verrucomicrobiaceae*, which are capable of producing various short-chain fatty acids (SCFAs) and may contribute to tumorigenesis by modulating the immune microenvironment (Yang et al., 2024; Situ et al., 2025). Wang and Zhu performed a Mendelian randomization (MR) analysis using pooled GWAS data on the skin microbiota of patients with HCC and found that *Firmicutes* exerted a protective effect against HCC (Wang and Zhu, 2024).

The human gut is a complex and dynamic microbial ecosystem. Previous studies have shown that microorganisms produce a wide range of metabolites that can alter their microenvironment and participate in diverse metabolic pathways (Visconti et al., 2019). To further investigate the relationship between biliary microbiota and disease, we used PICRUSt2 to predict the metabolic functions of microorganisms in group T. PICRUSt2 analysis revealed that KEGG pathways in group T were enriched in lipid metabolism and energy synthesis, including amino acid biosynthesis, ribosome-related pathways, 2-oxocarboxylic acid metabolism, and phenylalanine, tyrosine, and tryptophan biosynthesis. This aligns with the metabolic reprogramming of tumor cells, which exhibit high biosynthetic flux and elevated energy demand. Amino-acid synthesis and catabolism anaplerotically feed the tricarboxylic acid (TCA) cycle, sustaining adenosine triphosphate (ATP) production and thereby supporting tumor cell survival. Previous studies have shown that in CCA, alterations in metabolites such as amino acids may promote cancer progression by influencing energy production (Prajumwongs et al., 2025). Moreover, amino acids play an important role in redox balance and tumor-associated immune responses (Lieu et al., 2020; Chen et al., 2024). Notably, tryptophan—an essential amino acid—has a pivotal role in cancer progression. Its metabolites, including kynurenine, indole-3-acetaldehyde, and indolyl sulfate, are frequently upregulated in tumor. Among these, kynurenine can promote tumor progression by suppressing antitumor immune responses, among other mechanisms (Abd El-Fattah, 2022). In addition to this, it has been shown that altered levels of kynurenine correlate with non-small-cell lung (Jia et al., 2015) and colorectal cancers (Damerell et al., 2025), that altered levels of 3-IAA correlate with overall survival in pancreatic cancers (Tintelnot et al., 2023). In addition, indoxyl sulfate can activate the aryl hydrocarbon receptor and Akt signaling pathways, inducing epidermal growth factor receptor (EGFR) expression and promoting colorectal cancer progression (Ichisaka et al., 2024).

Altered metabolism of fatty acids in tumors is of wide interest, they are a fuel source for energy production in tumor cells in addition to being a structural component of the membrane matrix. The lipid-derived metabolite carnitine facilitates mitochondrial fatty-acid transport in tumor cells, enhancing β-oxidation and ATP production to support cell survival (Koundouros and Poulogiannis, 2020). Padthaisong et al. investigated potential mechanisms underlying recurrent CCA and reported that most lipids, including triglycerides (TGs) and phosphatidylcholines (PCs), were upregulated in recurrent CCA. Moreover, cancer stem-like cell (CSC) biomarkers such as CD44v6 are also involved in lipid uptake and are associated with recurrence-free survival (Padthaisong et al., 2021). Previous studies have also shown that dysregulated lipid metabolism is associated with poor prognosis in breast cancer (Abdelzaher and Mostafa, 2015) and in HCC (Morita et al., 2013).

Spearman correlation analysis revealed a positive linear association between chenodeoxycholic acid (CDCA) and *Enterococcus*. CDCA, a primary bile acid, is involved in metabolic pathways such as bile acid biosynthesis and bile secretion, and alterations in its levels are closely associated with obstructive biliary disease. Krupa et al. further confirmed the relationship between biliary obstruction and bile acid salts (Krupa et al., 2021). The farnesoid X receptor (FXR), a bile acid–activated nuclear receptor, is downregulated in human CCA cells compared with normal cholangiocytes *in vitro*. Moreover, CDCA inhibits CCA cell proliferation through FXR activation (Erice et al., 2018; Di Matteo et al., 2019). Our study demonstrated a significant positive correlation between CDCA and *Enterococcus*, and their combined assessment may improve the diagnostic performance for CCA in future studies.

It has to be admitted that our study has some limitations. First, this study is exploratory in nature and does not involve formal sample size calculation. The sample size was determined based on existing literature and practical considerations. Given the relatively small sample size, it is necessary to increase the sample size in future studies to validate and expand upon these preliminary results. Second, 16S rRNA sequencing provides limited taxonomic and functional resolution. Shotgun metagenomic sequencing may allow strain-level characterization and more comprehensive functional profiling, and key metabolomic findings should be mechanistically validated through targeted in vitro experiments. Future studies could explore the effects of fecal microbiota transplantation (FMT) or probiotic interventions in CCA models and integrate multi-omics data, such as metatranscriptomics, to construct a more comprehensive regulatory network for CCA.

## 5 Conclusion

This study integrated 16S rRNA sequencing and metabolomics to characterize the unique microbial community structure and metabolic profile of patients with CCA. Our findings suggest that microbiome–metabolite interactions may contribute to CCA development through mechanisms involving inflammation, oxidative stress, and energy metabolism. Our study provides potential microbial and metabolic markers for the early diagnosis of CCA and lays a theoretical foundation for the development of novel therapeutic strategies. Future studies with larger cohorts and experimental validation are warranted to further clarify the clinical significance and translational potential of these findings.

## Data Availability

The names of the repository/repositories and accession number(s) can be found at: 16S rRNA sequencing data are stored in the NCBI database under accession number PRJNA1282669, https://www.ncbi.nlm.nih.gov/; metabolite data are stored in the NGDC database under project number PRJCA042401, https://ngdc.cncb.ac.cn/.
