# Supplementary material for: Association of biliary microflora dysbiosis with cholangiocarcinoma: a single-center study
Source: Front Microbiol. 2025 Nov 3;16:1666272. doi: 10.3389/fmicb.2025.1666272 (PMC12622893; doi:10.3389/fmicb.2025.1666272)
Supplement: Supplementary file 1 [file Data_Sheet_1.docx]

Supplementary Material

## Supplementary Figures


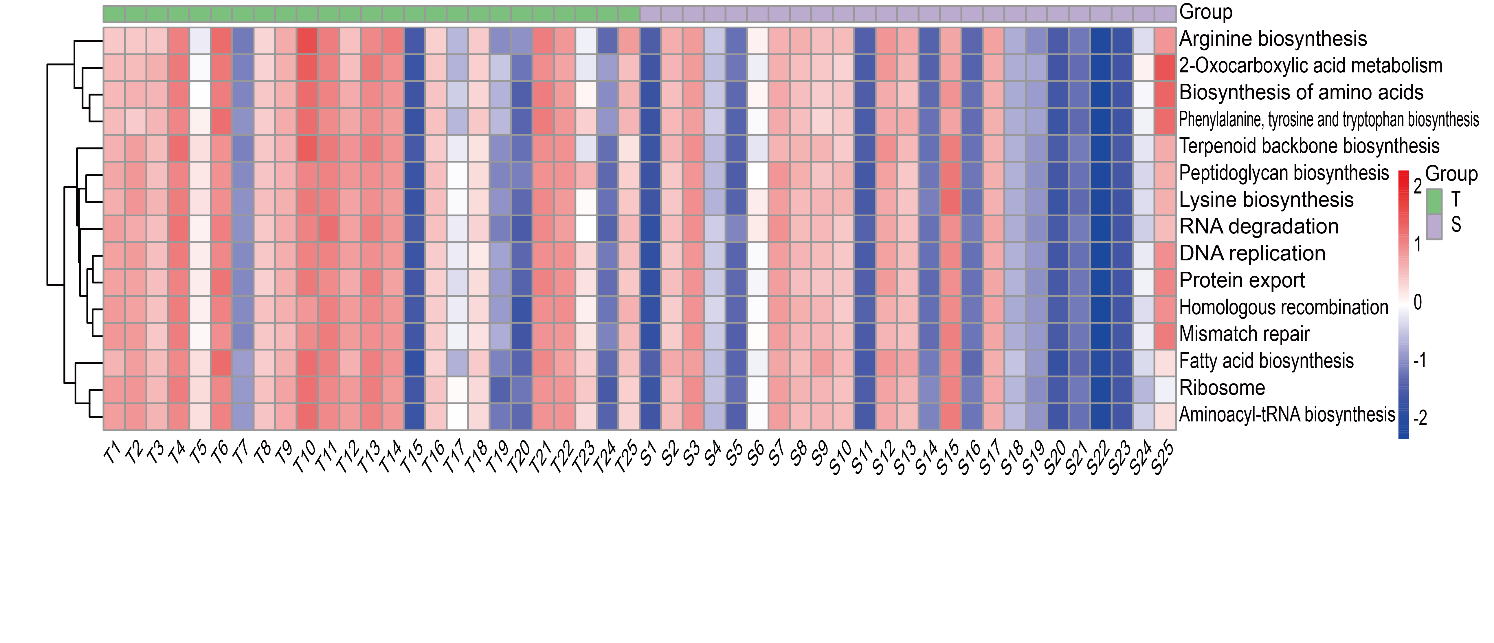


**Supplementary Figure1.** Heatmap of KEGG Difference Result Clustering, light green represents group T, light purple represents S, and the colours indicate the significance of the p-value.


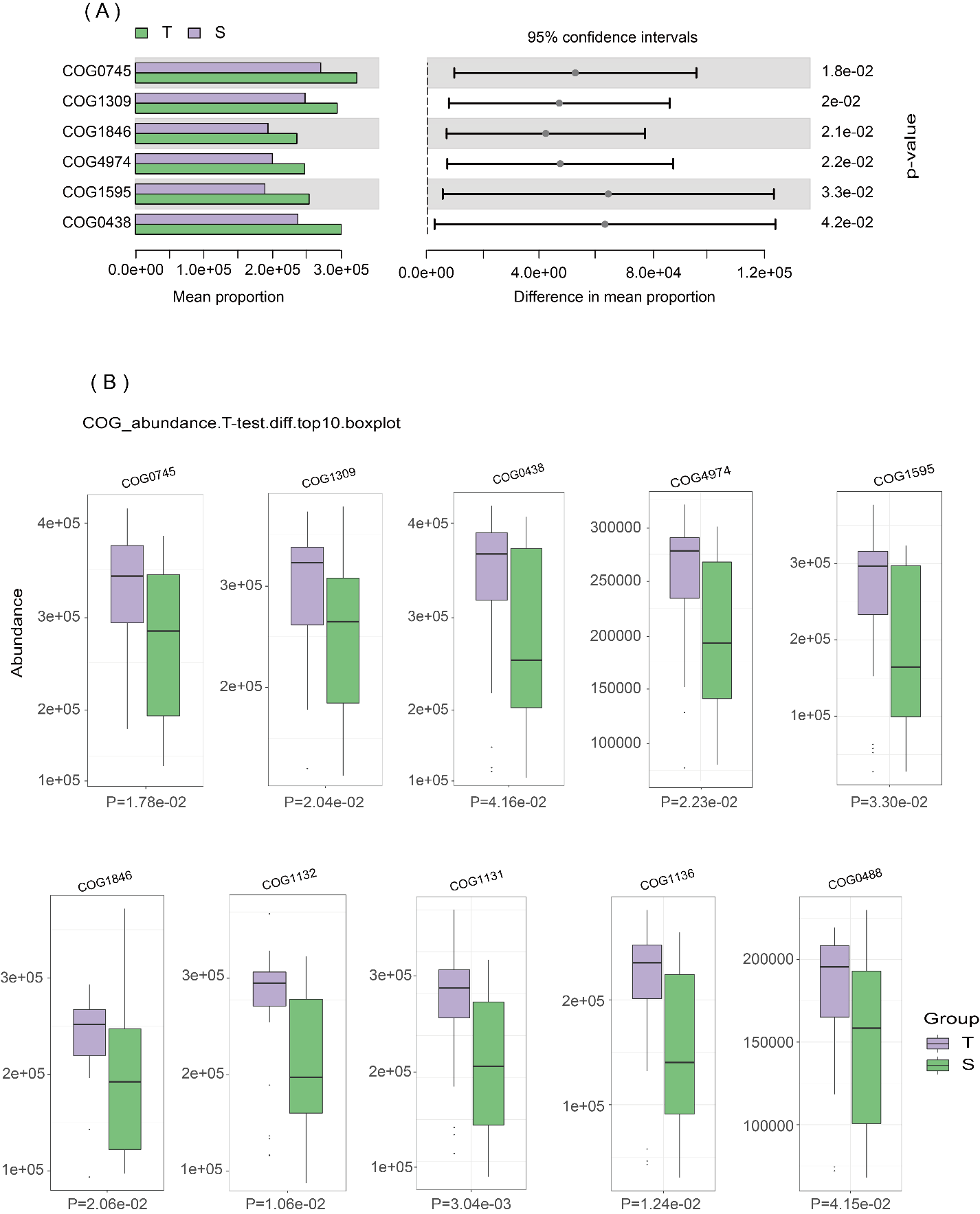


**Supplementary Figure2.** COG Differential results chart. **(A)** COG difference results bar graph, bars show average abundance of pathways in each group, light green represents group T, light purple represents S. **(B)** COG Differential Results Boxplot, light green represents group S, light purple represents T.


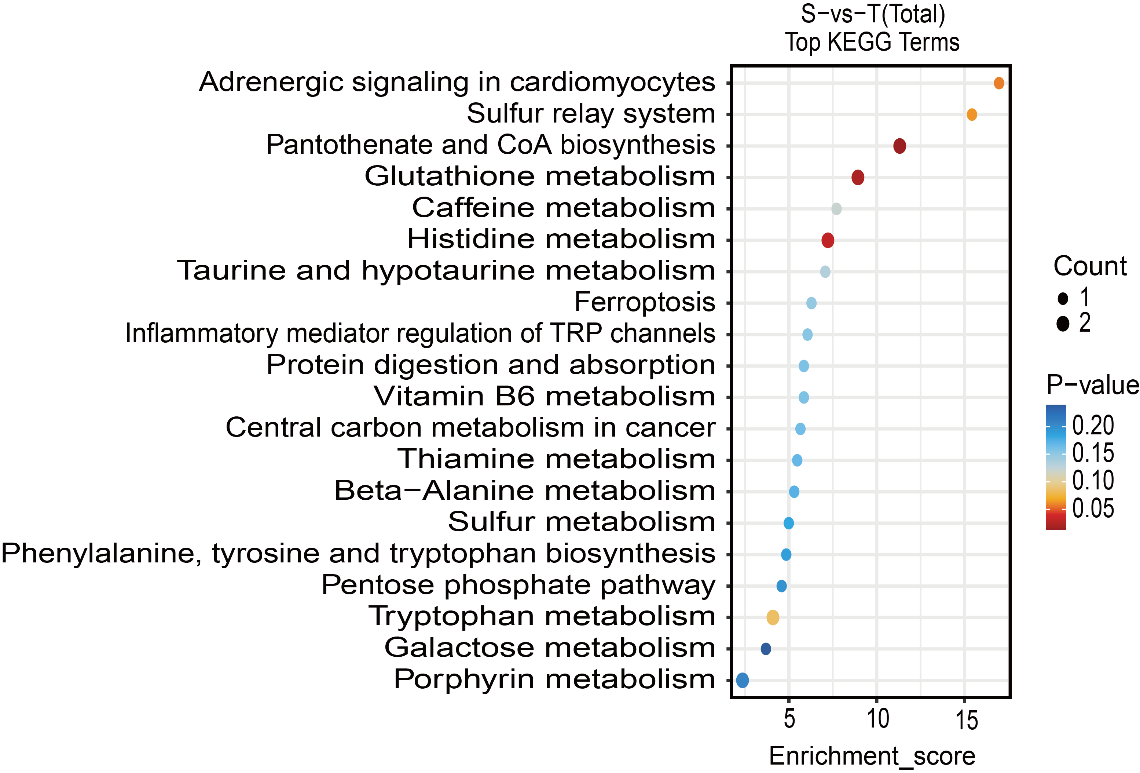


**Supplementary Figure3.** KEGG enrichment analysis: the horizontal coordinate is the enrichment score, the vertical coordinate is the top20 pathway information, the size of the bubbles reflects the number of differential metabolites in each pathway, and the colours indicate the significance of the p-value.


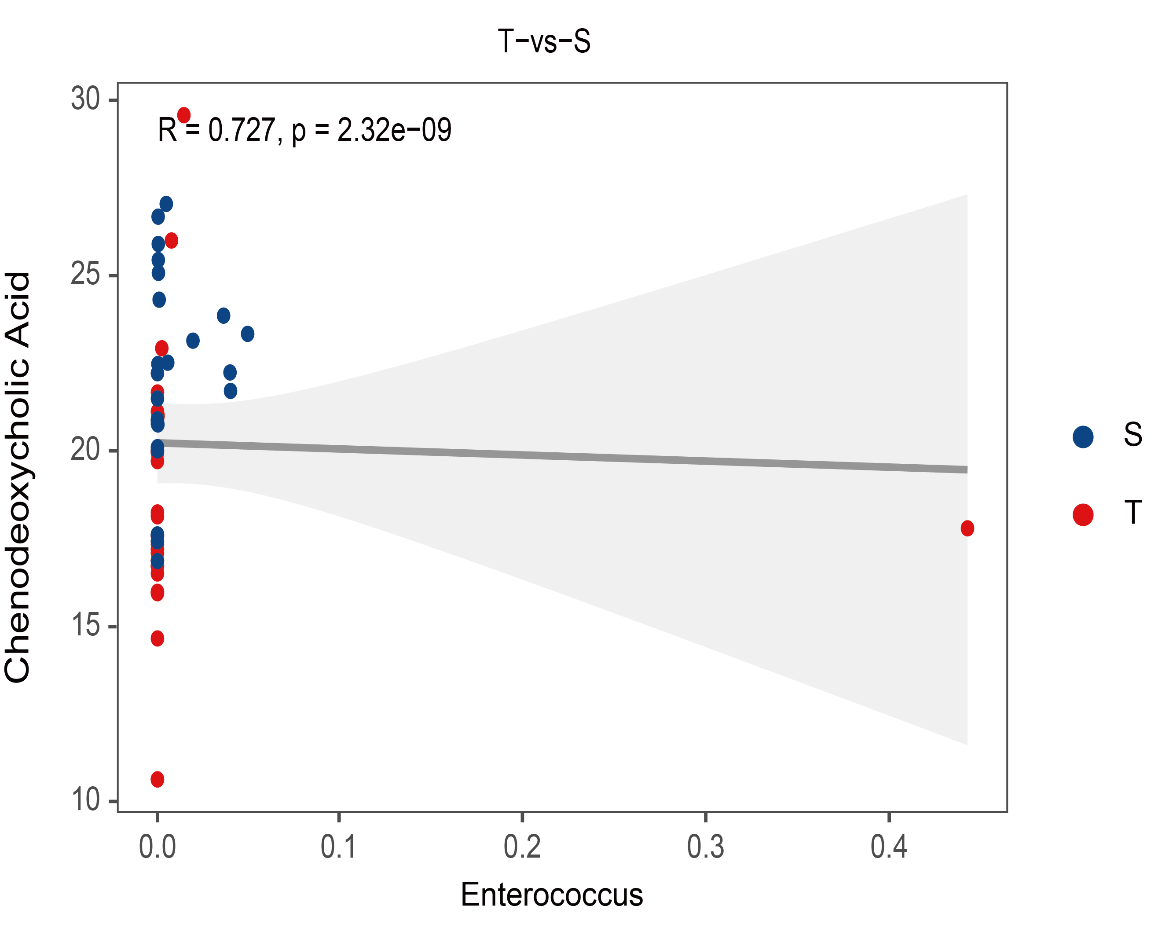


**Supplementary Figure4.** The scatter plot of the correlation between metabolite Chenodeoxycholic Acid and microorganism Enterococcus shows a correlation coefficient of R=0.727 and p=2.32e-09 within a 95% confidence interval. The color of the dots represents the grouping, with blue indicating the stone group and red indicating the tumor group.
